# Serum Vaspin Levels Are Associated with the Development of Clinically Manifest Arthritis in Autoantibody-Positive Individuals

**DOI:** 10.1371/journal.pone.0144932

**Published:** 2015-12-15

**Authors:** Karen I. Maijer, Elena Neumann, Ulf Müller-Ladner, Daniël A. C. A. D. Drop, Tamara H. Ramwadhdoebe, Ivy Y. K. Choi, Daniëlle M. Gerlag, Maria J. H. de Hair, Paul P. Tak

**Affiliations:** 1 Division of Clinical Immunology and Rheumatology, Academic Medical Center/University of Amsterdam, Amsterdam, the Netherlands; 2 Department of Internal Medicine and Rheumatology, University of Giessen, Kerckhoff-Klinik, Bad Nauheim, Germany; University Hospital Jena, GERMANY

## Abstract

**Objectives:**

We have previously shown that overweight may increase the risk of developing rheumatoid arthritis (RA) in autoantibody positive individuals. Adipose tissue could contribute to the development of RA by production of various bioactive peptides. Therefore, we examined levels of adipokines in serum and synovial tissue of subjects at risk of RA.

**Methods:**

Fifty-one individuals positive for immunoglobulin M rheumatoid factor (IgM-RF) and/or anti-citrullinated protein antibodies (ACPA), without arthritis, were included in this prospective study. Levels of adiponectin, vaspin, resistin, leptin, chemerin and omentin were determined in baseline fasting serum samples (n = 27). Synovial tissue was obtained by arthroscopy at baseline and we examined the expression of adiponectin, resistin and visfatin by immunohistochemistry.

**Results:**

The development of clinically manifest arthritis after follow-up was associated with baseline serum vaspin levels (HR1.5 (95% CI 1.1 to 2.2); p = 0.020), also after adjustment for overweight (HR1.7 (95% CI 1.1 to 2.5); p = 0.016). This association was not seen for other adipokines. Various serum adipokine levels correlated with BMI (adiponectin r = -0.538, leptin r = 0.664; chemerin r = 0.529) and systemic markers of inflammation such as CRP levels at baseline (adiponectin r = -0.449, omentin r = -0.557, leptin r = 0.635, chemerin r = 0.619, resistin r = 0.520) and ESR (leptin r = 0.512, chemerin r = 0.708), p-value<0.05. Synovial expression of adiponectin, resistin and visfatin was not associated with development of clinically manifest arthritis.

**Conclusions:**

In this exploratory study, serum adipokines were associated with an increased inflammatory state in autoantibody-positive individuals at risk of developing RA. Furthermore, serum vaspin levels may assist in predicting the development of arthritis in these individuals.

## Introduction

Rheumatoid arthritis (RA) is a systemic autoimmune disease, characterized by synovial inflammation in multiple joints leading to joint damage and disability. The etiology of RA, though not completely understood yet, is considered multifactorial and genetic factors as well as various environmental and life style risk factors are considered to be involved. During recent years the incidence of RA has increased [1, 2]. The cause of this increase is not known, but it appears likely that environmental or life style factors account for this increase in a relatively short period of time. As the prevalence of obesity has increased dramatically, obesity may be an important life style risk factor in the development of RA [3]. However, the reporting of the potential influence of obesity on the development of RA has shown inconsistencies in cross-sectional studies [4–6]. We found in a prospective study in autoantibody positive subjects at risk of developing RA that after a median of 27 months follow up the overall arthritis risk was increased from 28% to 60% in individuals with a smoking history combined with overweight [7]. In contrast, the risk of developing arthritis in never smokers with normal weight was only 2%. The identification of obesity as a risk factor for the development of RA was supported by a larger prospective study [8].

Obesity is associated with a chronic inflammatory state. The most abundant cell type in adipose tissue is the adipocyte, but it also contains endothelial cells, fibroblasts, leucocytes and macrophages, which may highly infiltrate the adipose tissue in case of obesity. Adipocytes are known to secrete several bioactive peptides called adipo(cyto)kines [9]. These peptides include, amongst others, adiponectin, leptin, resistin, vaspin and visfatin. It is important to note that these peptides are not exclusively derived from adipose tissue, but may also be produced by for example macrophages at other sites. Furthermore, many other cytokines, such as tumour-necrosis factor (TNF), interleukin 1 (IL-1), IL-6 and monocyte chemotactic protein 1 (MCP-1) can be produced by the adipose tissue.

Serum levels of adipokines are higher in RA patients compared to healthy controls and non-RA controls and are related to disease activity [10–13]. Also in the synovial fluid and synovial tissue of RA patients adipokines are increased compared to non-RA controls [13–15]. Interestingly, adipose tissue obtained from the joint of RA patients can produce both pro- and anti-inflammatory cytokines as well as adipokines. Factors secreted by the RA articular adipose tissue can also stimulate fibroblast like synoviocytes (FLS) to produce pro-inflammatory cytokines [16]. Taken together, these observations suggest that adipokines produced by adipose tissue may play a role in the disease process in RA.

We hypothesized that adipokines may have a role in the development of RA during the preclinical stage of the disease. In this exploratory study, we examined serum levels and synovial expression of adipokines in autoantibody-positive individuals at risk of developing RA and evaluated their association with the subsequent development of RA.

## Patients and Methods

### Study subjects

Between June 2005 and September 2012 we included 51 individuals who were positive for immunoglobulin M rheumatoid factor (IgM-RF) and/or anti-citrullinated protein antibody (ACPA) and had either arthralgia and/or a positive family history for RA, but who did not present with arthritis (as determined by an experienced rheumatologist) [7]. Furthermore, they can be classified as having phase c (systemic autoimmunity associated with RA) with or without phase a (genetic risk factors for RA) and phase d (symptoms without clinical arthritis) in the development up to RA according to the recommendations of the EULAR Study Group for Risk Factors for RA [17]. Individuals were excluded if they had a history of arthritis, or if they had used disease-modifying antirheumatic drug (DMARD) therapy or corticosteroids for inflammatory joint complaints. In the current study, only individuals were included of whom fasting serum samples and/or synovial tissue was available. The study was approved by the Medical Ethics Committee of the Academic Medical Center/University of Amsterdam (AMC) and performed according to the Declaration of Helsinki. All patients gave written informed consent.

### Study design

At baseline, demographics were collected and the following clinical and laboratory parameters were obtained: 68 tender joint count (TJC-68); 66 swollen joint count (SJC-66); patient’s visual analogue scale (VAS) for pain (scale 0–100mm); body mass index (BMI) (overweight was defined as a BMI ≥25kg/m^2^ according to the World Health Association (fact sheet n°311)); IgM-RF levels using IgM-RF ELISA (Sanquin, Amsterdam, the Netherlands (upper limit of normal (ULN) 12.5IU/ml)) until December 2009 and thereafter using IgM-RF ELISA (Hycor Biomedical, Indianapolis, IN (ULN 49IU/ml)); ACPA using anti-citrullinated cyclic peptide (CCP)2 ELISA CCPlus (Eurodiagnostica, Nijmegen, the Netherlands (ULN 25kAU/l)); erythrocyte sedimentation rate (ESR (mm/hr)); serum levels of C-reactive protein (CRP (mg/L)). Yearly study visits were performed and for individuals who were suspected of having developed arthritis an additional visit was performed at which the presence of arthritis (clinically manifest joint swelling) was independently assessed by two investigators (MH and DG or IC and DG).

### Serum adipokines

A baseline serum sample after overnight fasting was taken in a subset of the autoantibody-positive individuals (n = 27) and stored at -80°C until further use. These samples were used to determine the levels of the following adipokines, using different comercially available ELISA kits; adiponectin (BioVendor, Human Adiponectin ELISA, High Sensitivity Sandwich ELISA (RD191023100)), leptin (R&D Systems, human Leptin Quantikin ELISA (DLP00)), chemerin (BioVendor, human Chemerin Sandwich ELISA (RD191136200R)), resistin (BioVendor, Human Resistin Sandwich ELISA (RD191016100)), omentin (Biovendor, human Omentin-1 Sandwich ELISA (RD191100200R)), and vaspin (AdipoGen, human Vaspin ELISA Kit 1a (AG-45A-0017)). Adipokine levels were measured in ng/mL.

### Mini-arthroscopic synovial tissue sampling

At baseline, all study subjects underwent mini-arthroscopic synovial tissue sampling of a knee joint as previously described [18, 19]. At least six specimens were collected for immunohistochemistry as described before to correct for sampling error [20]. The synovial tissue was snap-frozen en bloc in Tissue-Tek OCT (Sakura Finetek Europe B.V., Alphen aan de Rijn, the Netherlands) immediately after collection. Cryostat sections were cut (5 μm each) and mounted on Star Frost adhesive glass slides (Knittelglass, Braunschweig, Germany). Sealed slides were stored at -80°C until further use.

### Immunohistochemistry

Synovial tissue sections were stained using the following antibodies: goat polyclonal anti-adiponectin (Acrp30; R&D Systems, Minneapolis, MN), mouse monoclonal anti-resistin (184305; R&D Systems, Minneapolis, MN) and mouse monoclonal anti-visfatin (P4D5AT; Enzo Life Sciences, Farmingdale, NY). Staining of adiponectin and resistin was performed using a two-step immunoperoxidase method. Staining of visfatin was performed using a three-step immunoperoxidase method, as previously described [21]. As a negative control, isotype–matched immunoglobulins were applied to the sections instead of the primary antibody.

The expression of synovial adipokines was analyzed by semi-quantitative analysis (SQA) by two independent observers (KM and AD). The expression of adipokines in synovial tissue was scored on a 5-point scale (range 0–4), as previously described [22]. A score of 0 represented minimal expression, while a score of 4 represented high expression. Minor differences between observers were resolved by mutual agreement.

The tissue sections were also stained for CD68 to detect macrophages, CD3 to detect T cells, and CD55 to detect FLS and analyzed by SQA, as described before [23].

### Statistical analysis

Categorical data were depicted as number (%) and continuous variables as median (interquartile range, IQR). To compare baseline characteristics between the individuals who did develop arthritis after follow-up and those who did not, Chi-square test or Mann-Whitney U test was used as appropriate. Bivariate correlations were analyzed using Spearman’s rank correlation test. Cox’s proportional hazard regression analysis was used to evaluate the association of serum adipokines (assessed as continuous variable) and synovial tissue adipokines (assessed as categorical variable 0–4) with arthritis development. Follow-up duration was defined as the time between inclusion in the cohort and the onset of clinically manifest arthritis, or between inclusion and April 2014 (censored). We performed both univariate analyses, and analyses adjusted for overweight.

All statistical analyses were performed using SPSS v19.0 software (IBM Corp., Armonk, NY). A P-value of <0.05 was considered statistically significant.

## Results

Baseline characteristics are depicted in Table 1.

**Table 1:** Baseline demographic and clinical characteristics for individuals who did not develop arthritis after follow-up and individuals who did.

Characteristics	No arthritis N = 39	Arthritis N = 12	P-value
Sex, female (n (%))	27 (69)	8 (67)	0.868
Age, years (median (IQR))	48 (35–53)	48 (42–56)	0.386
CRP, mg/L (median (IQR))	2.0 (1.0–5.7)	3.4 (1.3–11.0)	0.208
ESR, mm/hr (median (IQR))	9 (2–15)	7 (5–14)	0.678
Patient VAS pain, mm (median (IQR))	28 (4–66)	52 (10–78)	0.298
TJC-68 (median (IQR))	2 (0–6)	2 (0–8)	0.856
Arthralgia present (n (%))	36 (92)	11 (92)	0.943
SJC-66 (median (IQR))	0	0	1.000
IgM-RF + (n (%))	21 (54)	8 (67)	0.433
ACPA + (n (%))	24 (62)	9 (75)	0.393
IgM-RF and ACPA + (n (%))	6 (15)	5 (42)	0.053
Smoking history, ever (n (%))	23 (59)	11 (92)	0.036
BMI (median (IQR))	24.6 (22.8–28.6)	27.6 (24.9–29.7)	0.053
BMI ≥25 kg/m^2^ (n (%))	18 (46)	9 (75)	0.083

Parameters are described as number (n (%)) or median (interquartile range, IQR) as appropiate. CRP = C-reactive protein; ESR = erythrocyte sedimentation rate; VAS = visual analogue scale; TJC = tender joint count; SJC = swollen joint count; IgM-RF = immunoglobulin M rheumatoid factor; ACPA = anti-citrullinated protein antibody; Smoking history = never smoked: 0 pack-years; ever smoked: >0 pack-years; BMI = body mass index; P-values < 0.05 in bold.

Of the 51 included individuals, 18 (35%) individuals were solely IgM-RF positive, 22 (43%) were solely ACPA-positive and 11 (22%) were positive for both autoantibodies. Twelve of the 51 (24%) individuals developed arthritis after a median follow up time of 22 (IQR 12–36) months. Of these 12 individuals who developed arthritis, 8 patients fulfilled the 2010 American College of Rheumatology and the European League Against Rheumatism (ACR/EULAR) criteria for RA at arthritis onset [24], 3 patients were initially classified as having unclassified arthritis but later on fulfilled the RA classification criteria, and 1 patient fulfilled the ACR classification criteria for osteoarthritis (OA) of the hand, but not for RA [25]. The median follow up time of the 39 (76%) individuals who did not develop arthritis was 26 (IQR 17–46) months (*classified as autoantibody-positive individuals at risk of developing RA)*.

In this cohort of autoantibody-positive individuals overweight was borderline significantly associated with development of arthritis (p = 0.083).

### Serum adiponectin, leptin and chemerin levels correlate with body mass index

Fasting baseline serum samples were available from 27 autoantibody-positive individuals (n = 9 developed arthritis, n = 18 did not develop arthritis). The median (IQR) concentrations of the different adipokines, expressed in ng/mL, were as follows: adiponectin 12792.0 (9399.8–17695.0), leptin 13538.0 (8932.9–25025.5), chemerin 152.7 (131.9–183.1), resistin 4.6 (3.9–6.1), omentin 720.0 (605.0–994.0) and vaspin 0.9 (0.4–1.6).

**Fig 1:**
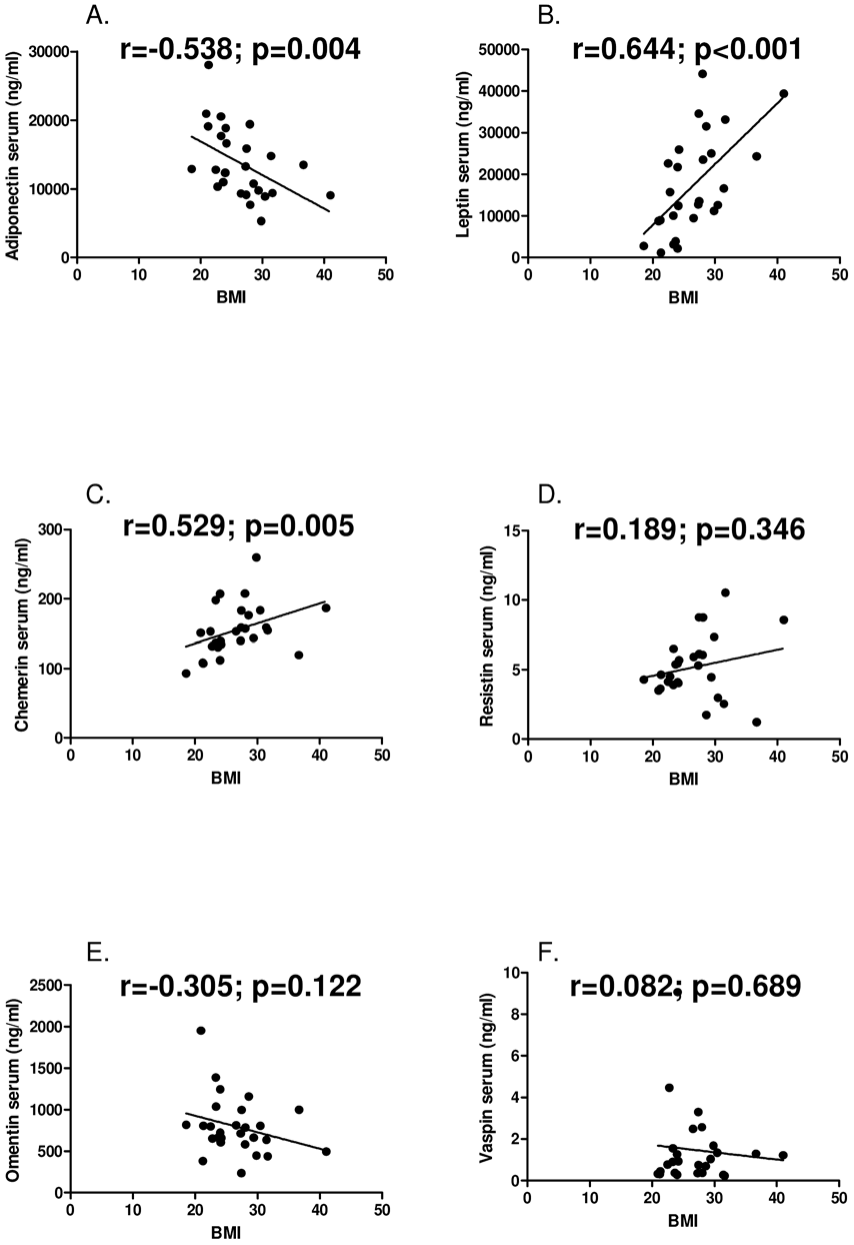
Correlations of serum levels of adipokines and body mass index. **A**, adiponectin **B,** leptin **C,** chemerin **D,** resistin **E,** omentin **F,** vaspin.

Serum adiponectin levels negatively correlated with BMI (r = -0.538; p = 0.004). Serum levels of leptin (r = 0.664; p<0.001) and chemerin (r = 0.529; p = 0.005) positively correlated with BMI. Serum levels of resistin (r = 0.189; p = 0.346), omentin (r = -0.305; p = 0.122) and vaspin (r = 0.082; p = 0.689) did not significantly correlate with BMI (Fig 1).

### Serum vaspin levels at baseline are associated with the development of arthritis after follow up

Serum vaspin levels were associated with the development of clinically manifest arthritis after follow up (HR1.5 (95% CI 1.1 to 2.2); p = 0.020), even after adjustment for overweight (HR1.7 (95% CI 1.1 to 2.5); p = 0.016) (Table 2). We did not observe an association between serum levels of adiponectin, leptin, chemerin, resistin, or omentin and the subsequent development of clinically manifest arthritis (see Table 2).

**Table 2:** Cox’s proportional hazard regression analysis for the association between serum adipokine levels and arthritis development.

**Variables in model Univariable**	**Hazard ratio (95% confidence interval)**	**P-value**
Vaspin	1.5 (1.1 to 2.2)	**0.020**
Adiponectin	1.0 (1.0 to 1.0)	0.352
Leptin	1.0 (1.0 to 1.0)	0.142
Chemerin	1.0 (1.0 to 1.0)	0.281
Resistin	1.2 (0.9 to 1.6)	0.155
Omentin	1.0 (1.0 to 1.0)	0.353
**Variables in model Multivariable**	**Hazard ratio (95% confidence interval)**	**P-value**
*Vaspin*	*1*.*7 (1*.*1–2*.*5)*	***0*.*016***
*BMI (≥25 vs ≤25 kg/m* ^*2*^)	*2*.*3 (0*.*5–10*.*7)*	*0*.*296*

BMI = body mass index; Univariate analysis for the association between serum adipokines and arthritis development. *Multivariate analysis for the association between serum vaspin and arthritis development*, *adjusted for overweight*. P-values < 0.05 in bold. Hazard ratio per unit increase in adipokine level, or for BMI ≥25 vs BMI ≤25 kg/m^2^

### Serum adipokine levels correlate with systemic markers of inflammation

Serum adiponectin (r = -0.449; p = 0.019) and omentin (r = -0.557; p = 0.003) levels negatively correlated with CRP levels while serum leptin (r = 0.635; p = <0.001), chemerin (r = 0.619; p = 0.001), and resistin (r = 0.520; p = 0.005) levels positively correlated with CRP levels. Serum levels of vaspin (r = 0.317; p = 0.115) did not correlate significantly with CRP levels (S1 Fig).

Serum levels of leptin (r = 0.512; p = 0.006) and chemerin (r = 0.708; p<0.001) correlated positively with ESR. Serum adiponectin (r = -0.141; p = 0.482), resistin (r = -0.047; p = 0.818), omentin (r = -0.079; p = 0.694) and vaspin (r = 0.063; p = 0.761) levels did not correlate significantly with ESR (S2 Fig).

### Synovial expression of adipokines is not associated with the development of arthritis after follow up

The synovial tissue of a range of 29 to 39 individuals could be included in the analyses for expression of the various markers by immunohistochemistry. This selection of individuals was based on the presence of sufficient quality of the tissue sections according to the strict quality control system based on the presence of an intimal lining layer.

**Fig 2:**
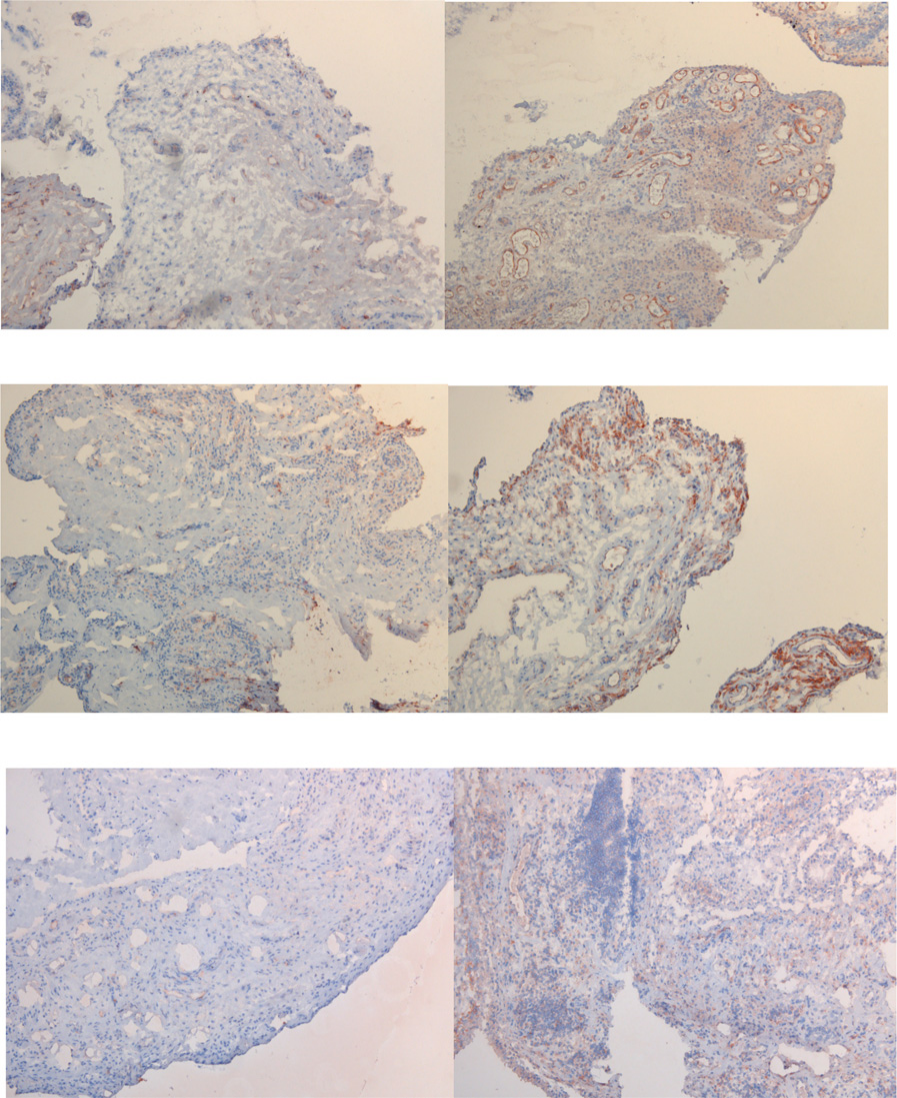
Baseline synovial expression of adipokines in autoantibody-positive individuals. In **A**, representative immunohistochemical staining of low expression of adiponectin (left) and of high expression of adiponectin (right). In **B**, representative immunohistochemical staining of low expression of resistin (left) and of high expression of resistin (right). In **C**, representative immunohistochemical staining of low expression of visfatin (left) and of high expression of visfatin (right). Magnification 100x.

Expression of adiponectin, resistin and visfatin in the synovium was not only observed in individuals who developed arthritis after follow-up, but also in those who did not. Expression of adiponectin was observed predominantly in the synovial sublining vasculature as well as in the surrounding sublining layers and to a much lesser extent in the intimal lining. Resistin expression was more observed in the synovial sublining layers compared to the intimal lining. Visfatin was expressed both in the intimal lining and the sublining layers, including some expression in the sublining vasculature (Fig 2). Synovial expression of adiponectin (df = 4; p = 0.567), resistin (df = 4; p = 0.924) and visfatin (df = 4; p = 0.706) was not associated with the development of clinically manifest arthritis.

### Association between serum and synovial levels of adipokines

Only for adiponectin and resistin were levels determined in serum as well as in synovial tissue. Serum levels of adipokines were not significantly related to synovial expression of these adipokines, consistent with the notion that the synovial tissue from clinically non-inflamed joints are not a major source of adipokines.

### Association between synovial adipokines and synovial markers of inflammation

There was not clear cut relationship between adipokine expression in the synovium and synovial markers of inflammation (data not shown). Of note, there was no question of arthritis in these joints, and there were only few inflammatory cells in the synovial tissue.

### Association between serum adipokines and synovial markers of inflammation

Previous work has suggested a positive relationship between synovial inflammatory cells and serum levels of adipokines in OA patients [26]. In our cohort of autoantibody-positive individuals at risk for RA we found a statistically significant relation between synovial inflammatory CD3 cells and serum leptin levels (p = 0.008), but otherwise we did not find a significant relationship between the presence of inflammatory cells in the synovium and serum levels of adipokines (data not shown).

## Discussion

Overweight appears to be a risk factor for the development of RA [7, 8] and is characterized by an increased volume of adipose tissue able to produce adipokines. This is the first study investigating serum levels and synovial expression of adipokines in autoantibody-positive individuals at risk of developing RA. In this exploratory study, we found a statistically significant association between serum levels of vaspin and the development of arthritis after follow up, also after adjustment for overweight. For the other serum adipokines examined we did not observe a relationship with arthritis development. Also, serum adipokine levels were correlated with BMI and systemic markers of inflammation, such as CRP and ESR. There was no clear cut relationship between adipokine serum levels and features of the synovium during the preclinical stage of the disease, supporting the view that in this phase of the disease joints are not a major source of adipokines.

The finding of vaspin levels in serum being associated with subsequent arthritis development may suggest a role for vaspin in the development of arthritis in these individuals at risk of RA. Apart from the fact that overweight appears to be a risk factor for RA, and vaspin is produced by adipose tissue, it is not immediately clear what the role of vaspin could be in the disease process. Various adipokines and cytokines produced by adipose tissue may play different and even opposing roles. It has been suggested that vaspin may have anti-inflammatory effects in the context of obesity-associated inflammation and cardiovascular disease [27–29]. Thus, vaspin could in fact play a role in the (failed) mechanisms aimed at resolution of inflammation [30]. Only very few studies investigated the role of vaspin in RA, describing elevated vaspin levels in serum and synovial fluid of RA patients when compared to healthy controls and OA patients, respectively [10, 31], but its role remains to be elucidated [32–34]. Consistent with our data, previous work showed no correlation between serum levels of vaspin on the one hand and levels of adipo(cyto)kines, acute-phase reactants and disease activity indices on the other hand in RA patients [10].

No association between serum adiponectin, leptin, chemerin, resistin, and omentin levels and the development of arthritis could be found, despite the fact that these adipokines are known to have anti- or pro- inflammatory effects in RA. Adiponectin, for instance, is the most abundant adipokine secreted from adipose tissue and exists in various isoforms with counteracting functions [32, 34]. Adiponectin can reduce TNF-induced monocyte adhesion as well as the expression of adhesion molecules and can therefore function as anti-inflammatory adipocytokine [35]. In RA, adiponectin expression has been found at higher levels in synovial fluid and synovial tissue compared to OA patients [14, 15]. Furthermore, serum adiponectin concentrations are higher in RA patients compared to healthy controls [12].

Another well studied adipokine is leptin and it is known to have various functions, including metabolic and immunoregulatory functions [9, 36–38]. In RA, serum levels of leptin were shown to be elevated compared to healthy controls [39].

The pro-inflammatory effects of resistin and chemerin have also been described [40, 41]. Resistin has been detected in RA synovial fluid at higher levels compared to non-inflammatory control patients [14] and expression of resistin and chemerin were higher in synovial tissue of RA patients compared to OApatients [13, 41].

Overall, the complex picture of several adipokines and their isoforms with different and even opposing effects could explain the fact that we did not observe a relationship between serum levels of the other adipokines examined (adiponectin, leptin, chemerin, resistin, and omentin) and subsequent development of arthritis.Adipokine levels are elevated in the synovial compartment of established RA patients, which suggests local production in the inflamed joint. We did not observe a relationship between synovial expression of adipokines and the subsequent development of arthritis. This can be explained by the fact that the joints in these individuals are not a major source of adipokines. The synovial tissue shows only minimal inflammatory cell infiltration during this stage of the disease [23].

We did find a positive correlation between serum levels of leptin, chemerin and resistin on the one hand and systemic markers for inflammation, such as ESR and CRP, on the other, which is in agreement with other reports in RA and metabolic syndrome l diseases [42–44]. Furthermore, leptin and chemerin also correlated positively with BMI, in line with other studies [45, 46]. In contrast, adiponectin and omentin levels correlated negatively with CRP and/or BMI in our study, which is consistent with the anti-inflammatory effects that have been described for adiponectin and with previous studies in obese and/or diabetic patients [47, 48]. Collectively, these data support the notion that overweight can lead to the secretion of both pro- and anti-inflammatory adipokines, collectively attributing to an altered inflammatory state.

There were some limitations of this study. The first limitation is the relatively small sample size; of 51 individuals fasting serum samples and/or synovial tissue was available, and only 12 (24%) individuals developed arthritis after follow up. Therefore, extensive multivariable analyses could not be performed. However, the results observed in our study provide the rationale for larger studies to build on these initial findings. The second limitation is that this study identified overweight using BMI, which is based on body weight regardless of its composition. Waist circumference and waist-to-hip ratio may reflect body fat more accurately.[49] Yet, these measures were not determined in our study.

In conclusion, this is the first, exploratory study in which serum adipokines have been associated with an increased inflammatory state and with overweight in autoantibody-positive individuals at risk of developing RA. In these individuals serum vaspin levels were associated with subsequent arthritis development, suggesting a role for vaspin in the development of arthritis in these individuals at risk of RA.

## Supporting Information

S1 Fig Correlations of serum levels of adipokines and C-reactive protein. **A**, adiponectin **B,** leptin **C,** chemerin **D,** resistin **E,** omentin **F,** vaspin.
(TIF)

S2 Fig Correlations of serum levels of adipokines and erythrocyte sedimentation rate. **A**, adiponectin **B,** leptin **C,** chemerin **D,** resistin **E,** omentin **F,** vaspin.
(TIF)
