# Supplementary figures and images for: Serum Vaspin Levels Are Associated with the Development of Clinically Manifest Arthritis in Autoantibody-Positive Individuals
Source: PLoS One. 2015 Dec 15;10(12):e0144932. doi: 10.1371/journal.pone.0144932 (PMC4682927; doi:10.1371/journal.pone.0144932)

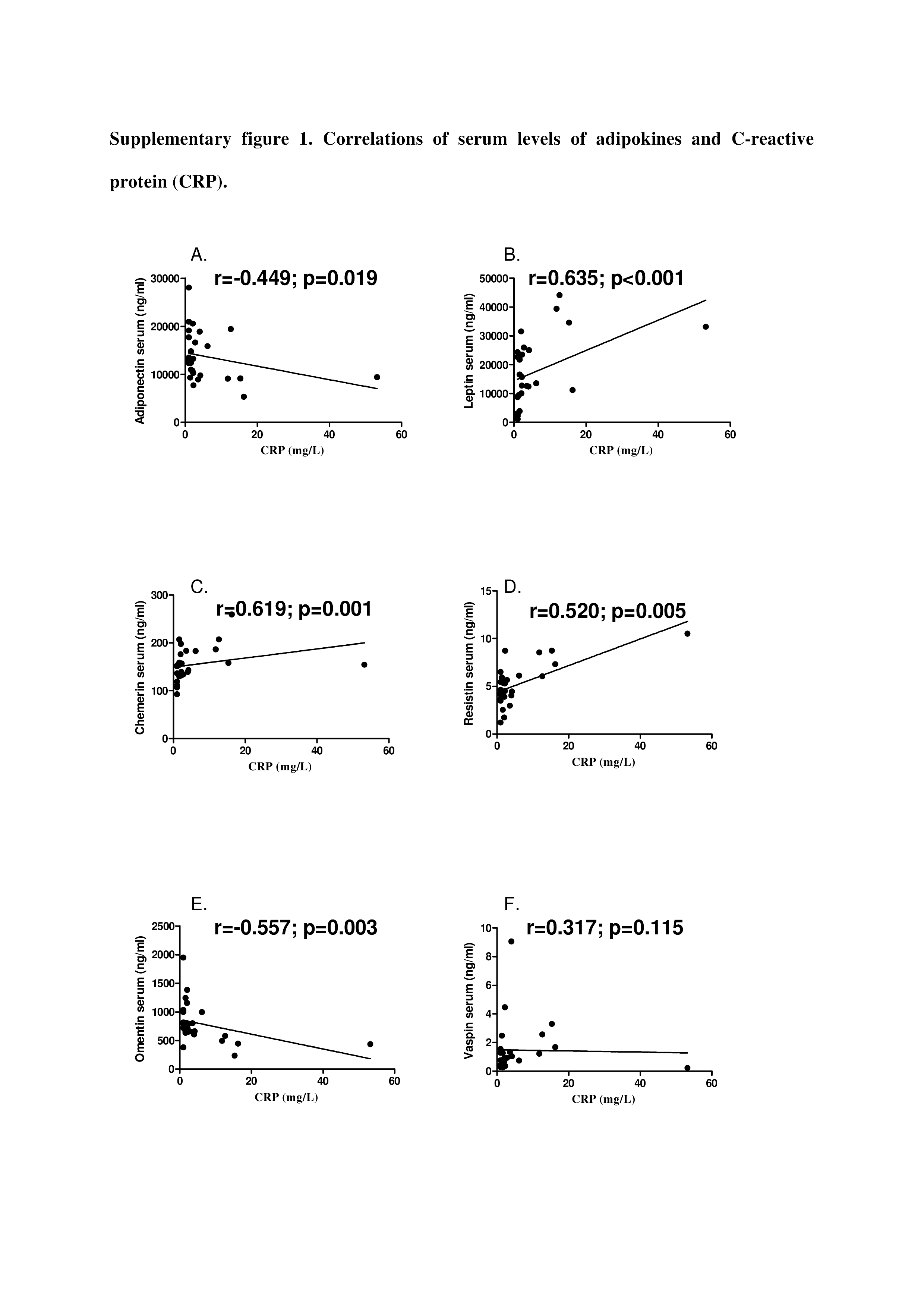

Supplement: S1 Fig — A, adiponectin B, leptin C, chemerin D, resistin E, omentin F, vaspin. (TIF) [file pone.0144932.s001.tif]

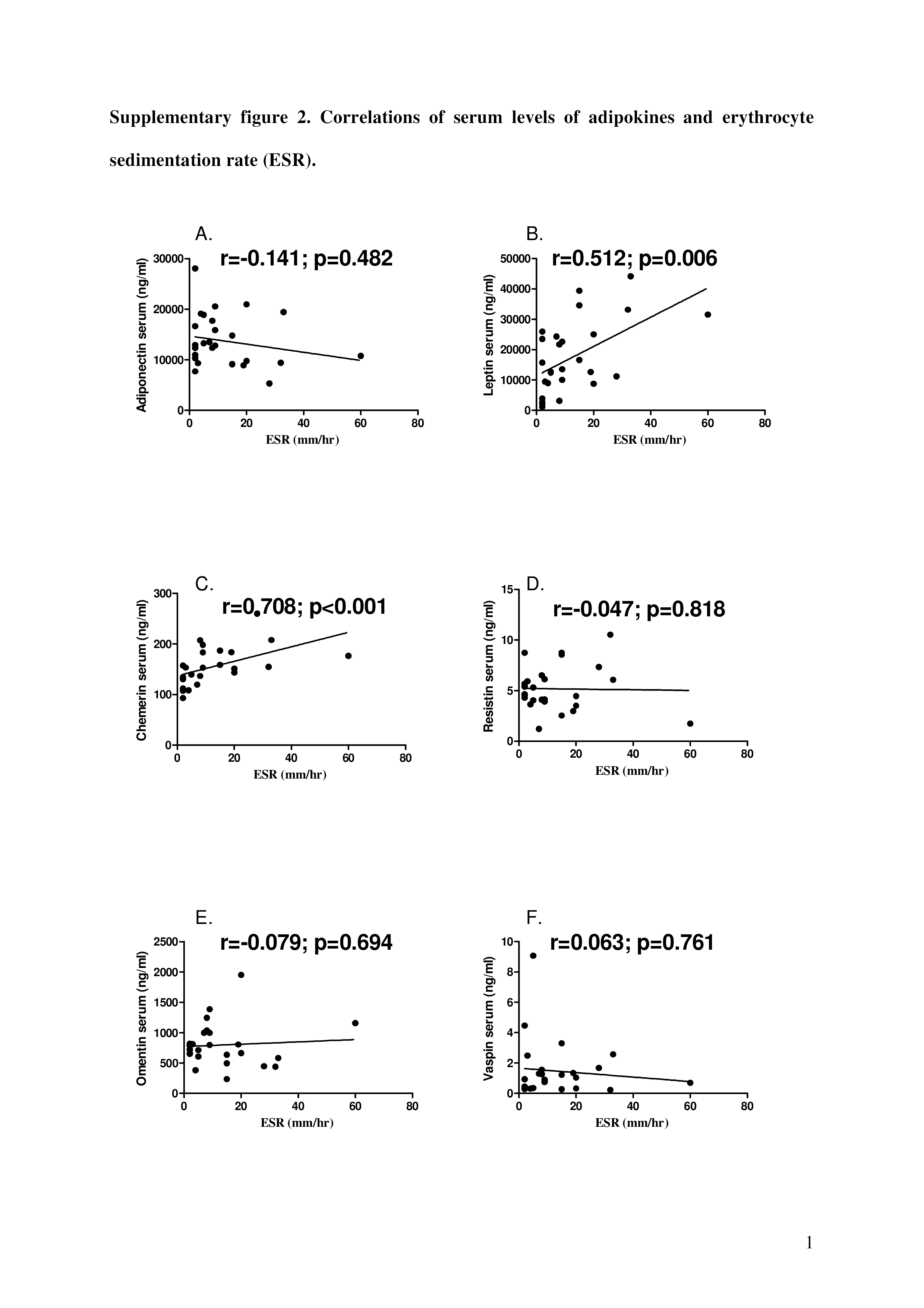

Supplement: S2 Fig — A, adiponectin B, leptin C, chemerin D, resistin E, omentin F, vaspin. (TIF) [file pone.0144932.s002.tif]
